# Pathways leading to success and non-success: a process evaluation of a cluster randomized physical activity health promotion program applying fuzzy-set qualitative comparative analysis

**DOI:** 10.1186/s12889-018-6284-x

**Published:** 2018-12-18

**Authors:** Christina Kien, Ludwig Grillich, Barbara Nussbaumer-Streit, Rudolf Schoberberger

**Affiliations:** 1 Department for Evidence-based Medicine and Clinical Epidemiology, Danube-University Krems, 3500 Krems an der Donau, Austria; 2 Center for Public Health, Department of Social and Preventive Medicine, Medical University Vienna, 1090 Vienna, Austria; 3 Department of Applied Psychology Work, Education, Economy, Faculty of Psychology, University of Vienna, 1010 Vienna, Austria

**Keywords:** Methods for implementation research, Qualitative comparative analysis, Process evaluation, Teacher professional development, Physical activity, School, Cluster RCT

## Abstract

**Background:**

Health promotion programs can only lead to improvements in health outcomes if they are effectively implemented. However, most studies assessing implementation success focus on only one condition, although more conditions influence this process. Therefore, evidence is scarce on what conditions play a role in successful implementation and how they interact. Hence, we aimed to identify which combinations of teacher and implementation process characteristics affected the *emotional and social school experience* (SCE) of pupils participating in a school-based health promotion program.

**Methods:**

This study was part of an effectiveness and process evaluation including 24 intervention and 27 control classes. We used fuzzy-set qualitative comparative analysis (fsQCA) to identify combinations of conditions that were associated with either an increase or no increase in the outcome *SCE* in comparison to the control group at 20 months post intervention. We deductively selected five conditions based on the Consolidated Framework for Implementation Research: teachers’ perceived self-efficacy, teachers’ expectations of the benefits of the intervention, teachers’ previous knowledge about the intervention, dosage of physical activity breaks, and quality of the implementation.

**Results:**

We identified five different pathways that led to no increase in the pupils’ outcome (parameters of fit: consistency 94%, coverage 66%). The combination of an *unsatisfying quality* of implementing the intervention and a *low previous knowledge* about the intervention showed the highest empirical relevance. Similarly, fewer physical activity breaks in combination with other conditions impeded the program’s success. Furthermore, we identified two different pathways characterizing ways to success (consistency: 81%, coverage: 52%). The most relevant combination was *good quality* implementation of physical activity breaks, implemented by teachers with a high *self-efficacy*, and a *good previous knowledge* about the intervention.

**Conclusions:**

QCA has potential for an in-depth analysis of complex interventions as it can rely on small to medium sample sizes and analyze pathways to success and non-success separately. The investigated program can be improved by considering the following suggestions: The quality of the implementation process should be monitored during the implementation phase, and regular feedback loops and learning opportunities for teachers should accompany a program. Clear recommendations regarding the dosage should be established.

**Trial registration:**

German register of clinical studies: DRKS00000622. Retrospectively registered: December 3, 2010, (http://www.drks.de/drks_web/setLocale_EN.do). Approved by the Ethics Committee of Lower Austria (GS4-EK-4/107–2010).

**Electronic supplementary material:**

The online version of this article (10.1186/s12889-018-6284-x) contains supplementary material, which is available to authorized users.

## Background

Physical activity has been associated with many health benefits in children and adolescents [1], such as improved mental health, well-being, and self-esteem [2–5], reduced age- and sex-adjusted body-mass index [6], and superior motoric competence [7]. However, most children and adolescents aged 5–17 years, internationally [8–11] and in Austria [12], do not achieve the recommended amount of 1 hour of daily physical activity of moderate to vigorous intensity [13]. Therefore, an important public health concern is to develop and disseminate effective interventions promoting physical activity in children and adolescents [14]. According to a recent socio-ecological approach [15], physical activity in children and adolescents should be addressed by pedagogical staff in settings where children live, learn, and play, such as schools. Furthermore, schools provide the opportunity to reach a large, unselected population consisting of children with diverse health-conscious behaviors as well as ethnic and socioeconomic backgrounds [16]. To increase the time children are physically active in the school setting [15], classroom-based physical activity interventions have become very popular recently (i.e. expansion of opportunities for children to be active). These interventions have revealed improvements in classroom behavior [17–19].

In complex interventions, it is always important not just to analyze the effectiveness of the program, but also to evaluate the quality of the implementation process, to investigate pathways of change, and to study context factors associated with outcome variation [20]. Implementation science is concerned with these questions. The implementation of interventions is defined as a “*specific set of activities designed to put into practice an activity or program of known dimension*” ([21], p.5). Process evaluation is a method to investigate these implementation issues and better understand the effectiveness of an intervention and its components [20]. A school setting, in particular, carries special challenges for implementing interventions: Schools are described as social, complex systems and interventions implemented in such systems may not always lead to predictable and intended outcomes, especially in non-researcher-implemented interventions [22, 23]. Different context factors in schools, such as a teachers’ individual preferences and opinions toward the intervention, as well as diverse practical challenges, such as time constraints, influence the way interventions are implemented [14]. Evidence shows that one important determinant factor is the perceived relative advantage associated with an intervention, which was shown to be positively associated with the dosage and the fidelity of the implementation of an intervention [24–26]. In addition, self-efficacy, a central variable in several individual behavior change theories, is positively associated with the intended implementation of the intervention and more positive health outcomes [4, 24, 27, 28]. Furthermore, the more intense the intervention and the better the implementation, the more often it leads to the intended health outcomes [17, 24, 28, 29].

However, most process evaluation studies in the field of prevention studies in general and physical activity trials in particular [24, 28] considered only one aspect for investigating the relationship between implementation (e.g. children’s participation in the intervention, intervention dose delivered) and outcome. In general, studies linking implementation process factors to the main outcomes at an individual level can assist in evaluating the effectiveness of the interventions. Furthermore, it allows for a better comparison of the results across studies and for improving the cumulative knowledge in the field of implementation science [30]. Currently, however, studies linking contextual factors, such as political, social, and organizational dimensions surrounding the implementation of an intervention [31], with health outcomes based on an implementation science framework are scarce [26].

We embedded our process evaluation in a cluster randomized controlled trial (cRCT) assessing an integrated school-based health promotion program for physical activity, called *Classes in Motion* [32]. The Swiss Model for Outcome Classification [33] assisted in developing clear goals of the intervention and clarifying the primary outcome. The program aimed to improve the primary school teachers’ practice of creating a positive and healthy learning atmosphere, which should affect the classroom climate. Therefore, the primary stakeholders (funder and program team) chose classroom climate as the primary outcome. This was part of the outcome category emotional and social school experience. In addition, we assessed 14 other outcomes grouped into the categories: emotional and social school-experience, self-reported physical activity, well-being, and attention performance. Overall, the cRCT showed small statistically significant differences in three different motor skills, but could not detect any relevant changes in the primary outcome or the other twelve outcomes [32].

The process evaluation focused on multiple influencing factors based on a theoretical model and explored which combinations of contextual factors and characteristics of the implementation process affected the participating primary school children’s *emotional and social school experience* including the primary outcome, the *classroom climate*. As the program did not lead to a statistically significant improvement of the primary outcome [32], a detailed analysis process should reveal which influencing factors led to an intervention and/or an implementation failure.

## Methods

This process evaluation was embedded in a cluster randomized controlled trial (cRCT). For the specific analysis of the process evaluation data, we applied qualitative comparative analysis (QCA), a research method developed by Charles C. Ragin [34] that is grounded in comparative social and political science. It perfectly suits studies with a small to intermediate number (e.g. 10–50) of cases [35] as this method was developed for analyzing case studies by broadening the understanding of the specific cases. This method investigates the relationships between *conditions* (i.e. explanatory variables in a model) and an outcome, relying on set theory. Set theory is a part of mathematical logic that studies sets (i.e. a collection of distinct objects, which is finally an object in its own right). QCA uses formal logic and Boolean algebra to identify conditions or combinations of conditions necessary and/or sufficient to reach an outcome [34]. The probabilistic approach used in regression analysis works differently and asks “what factor, *holding all other factors constant*, at each factor’s average, will increase (or decrease) the *likelihood of an outcome*” ([35], p. 202). We chose QCA as the method of analysis, as we investigated a small number of cases and aimed to answer a question related to the combinations of conditions and not related to the identification of the independent influence of a variable. Furthermore, we were interested, in whether different combinations of causal conditions could lead to the same outcome (i.e. causal complexity, [34], p. 23). For example, teachers possessing strong self-efficacy implementing the intervention with high quality and quantity could produce a similar positive outcome as teachers believing in the benefits of the intervention and implementing it with high quality and quantity. QCA effectively helped to identify combinations of conditions associated with larger patient weight-loss outcomes in a weight management program [36], with higher patient enrollment in cancer treatment trials [37], and with the identification of capacity elements resulting in achieving public health program objectives [38].

**Fig. 1:**
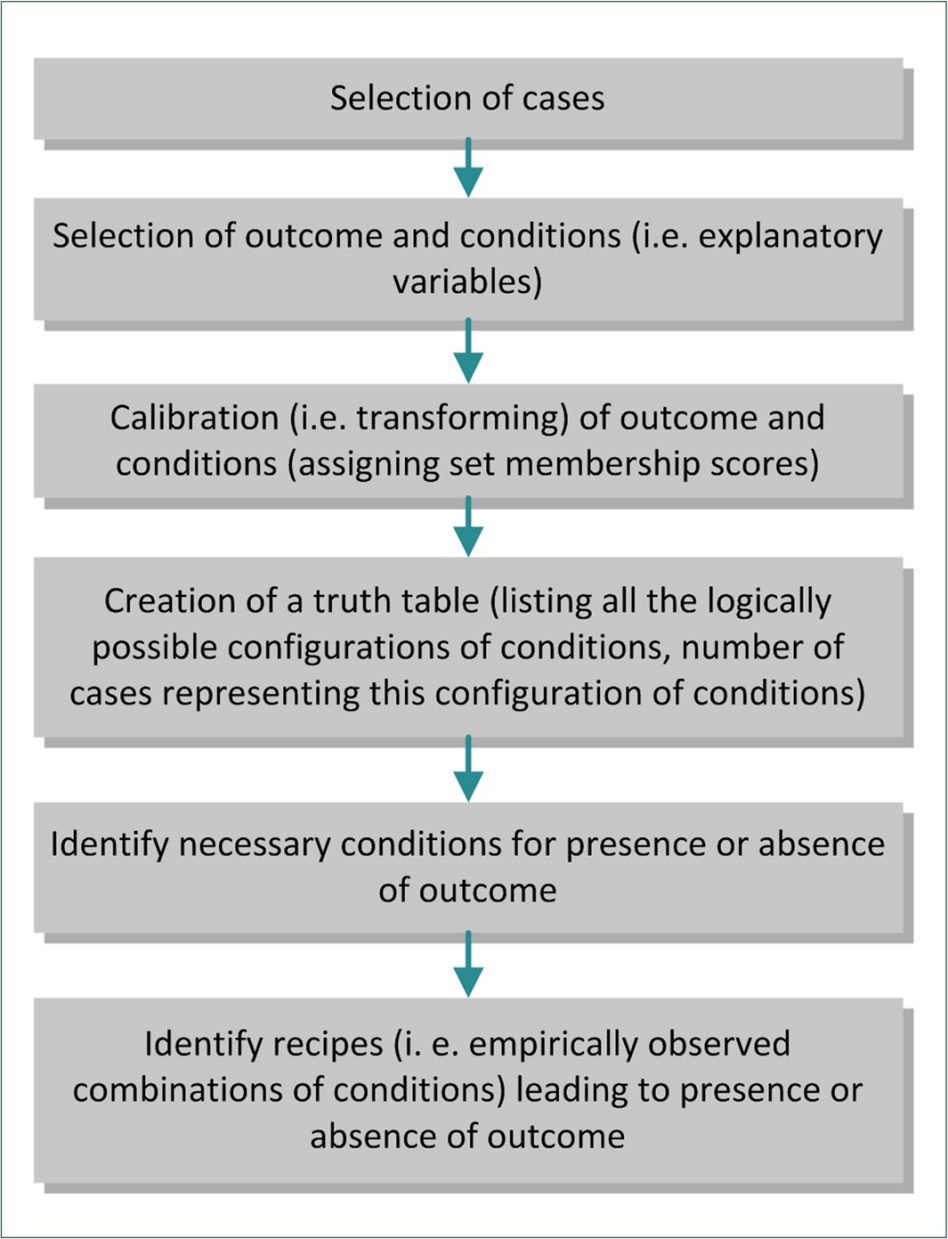
Description of data processing and analysis steps – fuzzy-set qualitative comparative analysis

We used a special type of QCA, the fuzzy-set qualitative comparative analysis (fsQCA), to identify relevant conditions for success and non-success of the implementation process. FsQCA assists researchers in classifying a gradual presence of a condition by assigning continuous membership values in a specific condition in the interval between 0.0 (non-membership) and 1.0 (full membership). Crisp set QCA (csQCA) is an alternative approach that treats cases as either fully in or out of a set (i.e. a condition or outcome is present or not present, respectively) based on definitions provided by the researchers. Therefore, only values of “0” or “1” are assigned to the conditions. In our study, our investigated conditions were assessed as continuous outcomes. To maintain the variance in the data, we chose fsQCA instead of csQCA. For a more detailed description of QCA in general and fsQCA in particular, see Kane et al. [35], Ragin [34], and Weiner et al. [37]. The data processing and analysis proceeded in six steps (Fig. 1).

### Design and participants

**Fig. 2:**
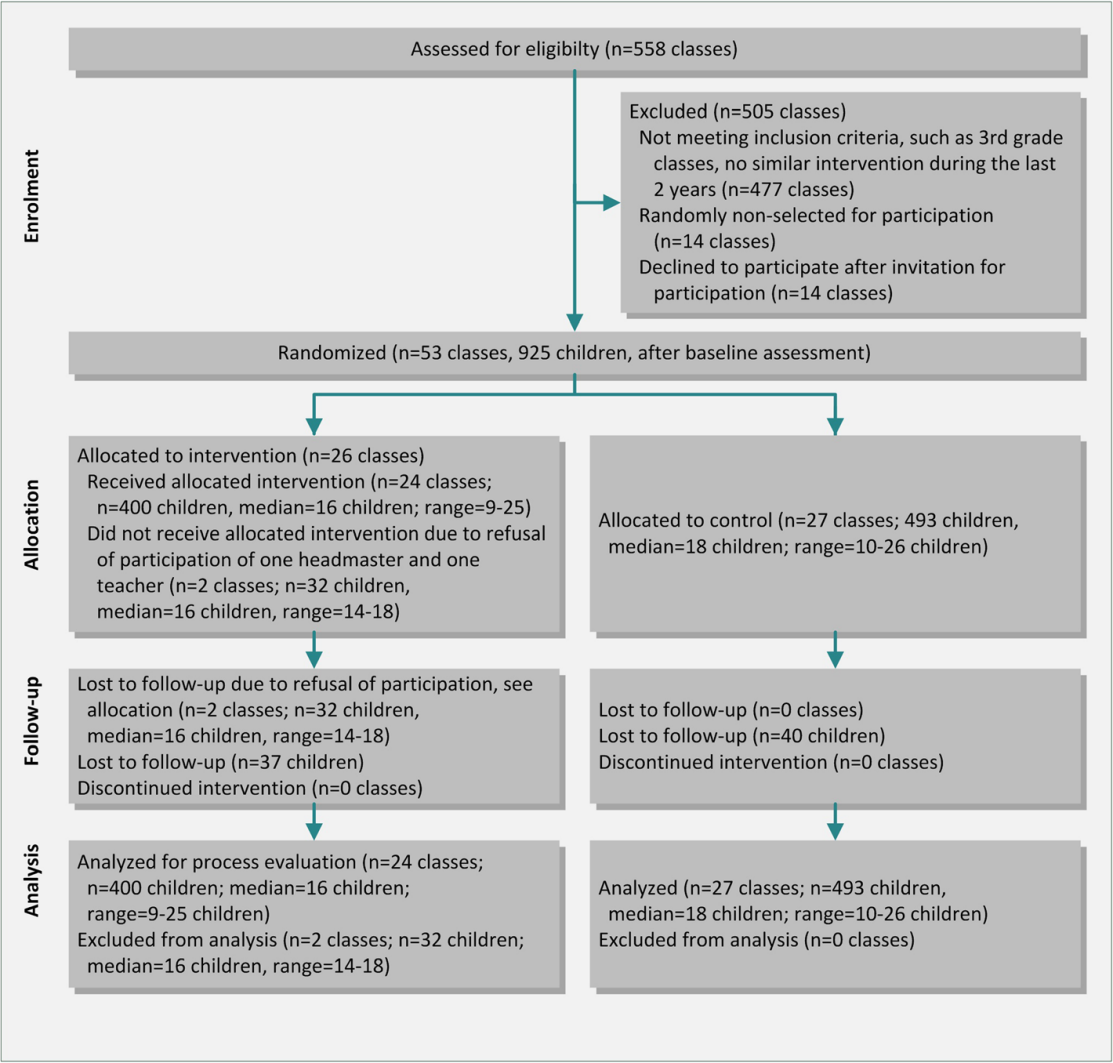
CONSORT flowchart describing progress of participants through the study

The cRCT with 20 months follow-up investigated the effectiveness of the school-based health promotion program *Classes in Motion* (German: *Bewegte Klasse*) for third-year elementary school children and took place in Lower Austria, a province of Austria. Overall, 26 classes were randomly allocated to the health promotion program and 27 classes to a waiting list control group in September 2010. As two classes in the intervention group discontinued their participation in the study after the baseline assessment and randomization, only 24 intervention school classes provided data for the follow-up assessment in April 2012. All classes were selected for the analysis process. Details on design and the results of the evaluation of the program’s effectiveness can be found elsewhere [32]. The flow of the school classes and participants through the study are depicted in Fig. 2, see also [32].

### Intervention

*Classes in Motion* follows a multiplier approach and aims to foster a teacher’s competency in creating a healthy and positive learning environment and integrating more physical activity into the curriculum to improve the pupils’ positive emotional and social school experience, increase their levels of physical activity, foster their motoric skills, and improve their well-being and attention. Overall, the trainers encouraged the teachers to include active breaks in the regular curriculum, which are short bouts of physical activity leading to a break from the normal course of the curriculum, and to include curriculum-focused active breaks that also comprise academic content [17]. The participating teachers received 20 on-the-job-training lessons of 50 min in length (i.e. sample training lessons to demonstrate how physical activity can be included in the classroom) over the course of 1.5 academic years (November 2010 – February 2012). Eight qualified health promotion specialists with diverse backgrounds, including teachers, psychologists, or outdoor trainers, taught these on-the-job-training lessons, which were directed toward the children and the teachers and focused on different topics covering active learning, team building, and social competence. Furthermore, teachers took part in two workshops (i.e. lectures on physical activity, and self-awareness exercises; overall, 16 sessions of 50 min duration each) on the effects of physical activity, learning theories, and practical didactical techniques. Moreover, they received additional information in a handout. The teachers in the waiting control group received the intervention only in the next school year after the follow-up assessment was conducted. Overall, knowledge and competence translation worked by 1) professional learning in the group, 2) one-to-one coaching, and 3) training materials for teachers to use in class. In Austrian primary schools, one teacher educates children on all subjects, except religion, in one specific class. During the course of the research process, five teachers went on maternity leave. All of the replacement teachers continued to participate in the program.

### Data collection

#### Outcome variable

As already mentioned, the primary stakeholders (funder and project team) chose c*lassroom climate* as the primary outcome. This outcome was grouped by the primary stakeholders into the outcome category *emotional and social experiences at school*. We measured this outcome category using three subscales of a reliable and validated questionnaire on *Emotional and Social Experiences at School of Elementary School Children*, FEESS 3–4) [39]: 1) *Classroom Climate* (11 items), 2) *Attitude towards School* (14 items), and 3) *Feeling Accepted by the Teacher* (13 items). We aimed to assess the influence of contextual factors of different classes. Therefore, the unit of our analyses were the classes participating in the intervention. Consequently, the *emotional and social experiences* of pupils were considered as improved when the difference between baseline and follow-up assessment was one standard deviation above the difference of the comparison group. To reduce the number of investigated outcomes, we counted one improvement in a subscale as an improvement in the outcome *emotional and social experiences at school*. To build an overall score of the three subscales is not considered a valid assessment tool [39]. Trained and blinded examiners conducted the baseline and follow-up assessment.

#### Conditions (influencing variables)

We concentrated on contextual factors from the *Consolidated Framework for Implementation Research,* CFIR [29, 40]. CFIR is considered to be a determinant framework, describing factors that have been shown in other implementation studies to act as barriers or enablers of the implementation process [41]. During the course of the process evaluation, we measured more variables, e.g. personal attributes of the teacher, and teachers perceived appropriateness of the training. Finally, we selected five conditions based on the following three criteria: fit to CFIR constructs, observed variation in the condition, and potentially modifiable through teacher training unlike fixed personal attributes, such as gender or age. We assumed that these conditions could be influenced, if teachers would learn more about the theoretical assumptions of the intervention and about how to implement the intervention. The third criteria did not apply to the condition *previous knowledge about the intervention*. The primary stakeholders (funder and program team) together with the researchers developed a logic model [33] for the intervention (see Additional file 1). These five conditions consisted of two aspects describing the *implementation process* domain (*executing: quantity* and *quality of the intervention*), one condition from the *intervention characteristics* domain (*relative advantage of the intervention*), and two conditions from the *characteristics of the individuals providing the intervention* domain (*self-efficacy and knowledge about the intervention*).

*The quantity (*i.e. *dosage) of the intervention* [24, 29] is an important determinant of diverse intended health outcomes [17, 28]. In our study, teachers reported the average number of minutes per day they were using physical activity in the classroom and the number of days in the last month they usually practiced physical activity during regular classes (filling out a paper-pencil questionnaire at the follow-up assessment, see Additional file 2). Finally, we calculated the average weekly amount of minutes of physical activity during regular classes.

*Quality of the implementation* process refers to how well the intervention has been carried out by the intervention providers [24, 29]. At the end of the first academic year and at the time of the follow-up assessment, the trainers rated the quality of the teacher-provided intervention on a three-point rating scale. Higher values indicated a higher quality of the provided intervention.

*Relative advantage* is described as the “stakeholders’ perception of the advantage of implementing the intervention versus an alternative solution” ([29], p. 6). Teachers answered eight items in a paper-pencil questionnaire regarding the expected benefits of physical activity during regular classes on a four-point rating scale ranging from “4 – strongly agree” to “1 – strongly disagree” (e.g. “Physical activity during classes improves children's concentration”, see Additional file 2).

*Teacher’s self-efficacy* measures the subjective belief in one’s capabilities to act in order to achieve certain intended implementation outcomes [42]. Teachers rated their self-efficacy in implementing the intervention by means of two items on a four-point rating scale ranging from “4 – strongly agree” to “1 – strongly disagree” in a questionnaire provided at the follow-up assessment. An example item reads as follows: “I am confident that I can implement active breaks effectively.”

*Knowledge about the intervention* is defined as the understanding of facts, strategies, and principles about the intervention [29]. We operationalized this condition as the sum of possibilities that teachers have heard and learned about this intervention before they participated in this program. This was self-assessed on a four-point rating scale ranging from “4 – strongly agree” to “1 – strongly disagree” in a questionnaire provided at the follow-up assessment (see Additional file 2).

### Condition and outcome set calibration

The most important step in fsQCA is to transform the relevant outcome and conditions into new variables based on decision and definition rules. For this so-called *calibration* process, three anchors for the outcome and the investigated conditions need to be defined: full inclusion in the set of the new variable (corresponds to a membership score of 0.95), the crossover point with maximum ambiguity (membership score of 0.5), and full exclusion from the set of the new variable (non-membership score of 0.05). Based on in-depth knowledge of the cases and use of existing theory or prior research, the anchors are usually defined, making sense of variation in the data [34]. The primary stakeholders (funder and program team) together with the researchers developed a logic model for the intervention that guided the development of the decision rules described below [33] and listed in detail in Table 1. The same anchors were used for investigation of the pathways leading to both success and non-success. The first author defined the specific anchors and afterwards the members of the research team provided feedback to the anchors. Additionally, as part of the process evaluation we conducted qualitative interviews with the teachers of the intervention group of approximately 30 min length at the end of the intervention. As we used the information from the qualitative interviews only to verify the extreme cases of the quantitative questionnaire regarding the condition *benefits of the intervention*, we describe this methodological step briefly. With the consent of the participants, we recorded the semi-structured interviews and transcribed them verbatim, and ensured anonymity. Before we started the calibration process, we read through all the interviews to become familiar with the cases.

- For calibrating the condition *dosage of the intervention,* we drew on arousal theories proposing an inverted U-shaped association between performance and arousal [43]. It is assumed that children aged seven to 10 years have the potential to concentrate for up to 20 min, after which a physical activity bout or relaxation activity is seen as necessary [44]. With 3 minutes of physical activity per academic hour, four academic hours per day, and 5 days a week, a total of 60 min of physical activity bouts per week should be recommended, defining the anchor for full membership. Providing physical activity breaks on average only once a day for 3 minutes was considered the anchor for non-membership.
- Concerning the *quality of the implementation* condition, we defined the anchor for full membership when the trainers rated a teacher’s ability to conduct an active curriculum with the best quality rating twice (at the end of the first year and at the end of the program). If a teacher achieved at least twice the worst quality rating, full non-membership was stated.
- We calibrated the condition *relative advantage* using the anchors 3.84 for full membership (i.e. allowing a minimum of skepticism regarding the benefits of the intervention, which means that one out of eight was rated 3.0) and 3.4 for full non-membership (i.e. at least five out of three items were rated 3.0). Overall, this reflects the evidence [24–26] that higher expectations regarding the benefits of the intervention would be associated with a greater enthusiasm for implementing the intervention.
- We assigned the highest possible achievable value for *perceived self-efficacy* as the anchor for full membership with 10.0, drawing on the evidence that higher values lead to profounder implementation processes and better intervention outcomes [4, 24, 27, 28, 45]. We defined full non-membership with 6.1 as the anchor, where teachers already expressed substantial concerns about their confidence to implement the intervention properly.
- For the condition *knowledge about the intervention*, we defined the anchors for full membership with 3.65, giving the possibility that teachers rating one item with 3.0 instead of 4.0 out of three items would still qualify for full membership. Therefore, teachers rating their knowledge as very high or almost very high qualified for full membership.
- Finally, regarding the investigated *outcome*, we specified that a class would be fully included in the outcome set for success if 25% of the children in that specific class showed an improvement in *emotional and social school experience*. This low success rate was chosen in relation to the overall small improvements in the intervention group. The further anchors were defined so as to achieve similarly large groups for full non-membership as for full membership*.* It was defined as fully non-membership if 9% of the children showed an improvement in *emotional and social school experience*. The crossover point reflecting maximum ambiguity in membership was defined as 16%.

**Table 1:** Calibration of Conditions and Outcome for Success^a^

Feature	Explanation	Method of data collection (computation, number of items~)	Full Membership	Crossover Point	Full Non-membership
O: SCE	Emotional and social school experience	pupils self-report via questionnaire	0.25	0.16	0.09
C: PAB	Physical activity breaks in minutes/school week (dosage)	teacher self-report via questionnaire (2 items)	60	40	15.1
C: QOI	Quality of implementation	trainer rating via observation (mean, 2 time points)	2.99	2.56	1.49
C: PSE	Perceived self-efficacy of implementing the intervention	teacher self-report via questionnaire (weighted mean, 2 items)	10	7.9	6.1
C: BOI	Expected benefits of the intervention for pupils	teacher self-report via questionnaire (mean, 8 items)	3.84	3.6	3.4
C: KAI	Knowledge about the intervention (topic covered during education and further training, self-organized learning)	teacher self-report via questionnaire (mean, 3 items)	3.65	2.8	2.34

Abbreviations: *BOI* benefits of intervention, *C* Condition, *O* Outcome, *KAI* knowledge about the intervention, *PAB* physical activity breaks (dosage), *PSE* perceived self-efficacy, *SCE*, school-experience, *QOI* quality of implementation

^a^The same anchors were used for analyzing pathways of non-success. The full non-membership anchors for defining success relate to the full membership anchors for defining non-success and vice versa

~ The possible range, different values can take, is listed in Table 2

### Data analysis

After completing the calibration process, we created a truth table that lists all logically possible combinations of causal conditions in rows (e.g. *recipes,* pathways), the number of cases showing the combination of causal conditions in question, and the row-specific *consistency* value [34]. Consistency refers to the extent to which cases that show the same combinations of causal conditions lead to the same outcome [34, 35]. In general, 2^k^ logically possible combinations are presented in a truth table, where *k* stands for the number of conditions. Next, we reduced the number of rows in the truth table to those recipes that were empirically observed (i.e. at least one case per row), and where different cases showing the same recipes revealed the same outcome in 80% of the observed cases (i.e. minimum consistency threshold). This threshold was chosen as per convention [34]. We used the software fs/QCA version 2.5 [46] to conduct the analyses. First, we analyzed the presence of necessary conditions for success and non-success. These are conditions that must be present for an outcome to occur (i.e. showing a superset relationship with the outcome set). In other words, necessary conditions are always present when the outcome is present [35, 47].

Second, we identified the recipes that led to success and non-success by excluding logically redundant recipes based on Boolean algebra [34, 37]. As an example, if a list of recipes describes two identical combinations of conditions leading to an outcome, with the exception that condition B is present in one row and not present in the second row, then condition B can be dropped because whether or not this condition is present, the rest of the combination of conditions leads to the same outcome [34, 35]. In general, the analyses reveal three different *solutions* (i.e. describing all recipes resulting from the logical minimization process of the truth table). These different solutions are all logically consistent with each other, but depend on the different handling of rows in the truth table without any empirical case. Such rows are known as *logical remainders*). The complex solution does not take any simplifying assumptions about logical remainders into account. The parsimonious solution is created automatically and considers all possible simplification processes, whether or not they are plausible. The intermediate solution uses assumptions of simplification processes postulated by the researcher [48]. That means, that during the analyses the researcher can state – based on theory or plausibility – that a certain condition will be associated with the outcome. Based on these assumptions, rows in the truth table which are empty and are showing no empirical cases, contribute to the logical minimization process of the truth table. Finally, to assess the goodness of fit for the solution, we report *consistency* and *coverage* parameters of the solution. “Consistency is the degree to which instances of the outcome agree in displaying the causal condition” ([34], p. 44). Consistency resembles the statistical significance in a correlational assessment of two variables [34]. “Coverage assesses the relevance of the necessary condition – the degree to which instances of the condition are paired with instances of the outcome” ([34], p. 44). For describing the coverage of a specific recipe of a solution, further parameters of fit are available. The *raw coverage* discloses the proportion of cases demonstrating the specific recipe. The *unique coverage* reveals the proportion of cases uniquely covered by a specific recipe.

## Results

On average, 16% of school children in 24 classes of the intervention group showed an improvement in the outcome *emotional and social school experience* (SCE) above one standard deviation in relation to children of the control group (Table 2). The implementation process of the intervention was characterized by a mean duration of approximately 50 min of weekly physical activity breaks in the classroom, ranging from 10 to 125 min and by a quality rating ranging from 1 to 3. In the following paragraphs, we present the results regarding the combinations of conditions explaining the non-improvement of children participating in the intervention in the primary outcome SCE, as this was the more common case. Afterward, we demonstrate the pathways leading to success.

**Table 2:** Descriptive Statistics

	Feature	n	Mean	SD	Minimum	Maximum	Possible Range
Outcome	SCE	24	0.16	0.10	0.00	0.41	0–100%
Condition	PAB	24	49.69	32.84	10.00	125.00	≥ 0
Condition	QOI	24	2.23	0.66	1.00	3.00	1–3
Condition	PSE	24	8.69	1.23	6.00	10.00	3–10
Condition	BOI	24	3.62	0.37	2.38	4.00	1–4
Condition	KAI	24	3.06	0.60	2.00	4.00	1–4

Abbreviations: *BOI* benefits of intervention, *KAI* knowledge about the intervention, *n* sample size, *PAB* physical activity breaks (dosage), *PSE* perceived self-efficacy, *SCE* emotional and social school experience, *SD* standard deviation, *QOI* quality of implementation

### Explaining pathways to non-success (non-occurrence of emotional and social school experience)

No single causal condition was identified as leading to non-success of the program. Overall, of the 32 logically possible combinations of causal conditions to explain non-success, 16 different combinations could be observed, revealing a high diversity of causal combinations (Table 3). Eight different combinations of causal conditions met the minimum frequency threshold of one case and the minimum consistency threshold of 0.80. The complex solution, the solution that does not apply any simplifying assumptions about logical remainders (i.e. the rows in the truth table without any empirical case), could be mathematically reduced to five different combinations of conditions, including a minimum of four conditions per row (results not displayed). For achieving the intermediate solution, as already mentioned above, we took the assumptions that low quality [24, 29] and quantity [17, 28] of the implementation process, teachers doubting their self-efficacy for implementing the intervention [4, 24, 27, 28], and seeing fewer benefits in the intervention [24–26] would lead to a non-improvement of pupils’ positive emotional and social school experience. Overall, five different recipes described not achieving a positive emotional and social school experience in pupils due to the intervention (Table 4).

**Table 3:** Truth Table Summarizing the Recipes for Non-Achieving Pupils’ Emotional and Social School Experience

Conditions					Outcome	n	Consistency
PAB	QOI	PSE	BOI	KAI	Sce		
0	0	0	1	0	1	1	0.995
0	0	0	0	0	1	1	0.994
0	1	0	0	0	1	1	0.994
0	0	0	1	1	1	2	0.975
1	0	1	1	0	1	3	0.963
0	1	0	1	1	1	1	0.929
1	0	0	0	0	1	1	0.923
1	1	0	1	1	1	1	0.839
0	1	1	0	1	0	1	0.807
1	1	1	1	0	0	1	0.750
1	0	0	1	1	0	2	0.718
0	0	1	0	1	0	2	0.676
1	1	1	0	0	0	1	0.660
1	0	1	0	1	0	1	0.658
1	0	1	1	1	0	2	0.614
1	1	1	1	1	0	3	0.470

Abbreviations: *BOI* benefits of intervention, *KAI* knowledge about intervention, *n* number of school classes showing a specific causal condition, *PAB* physical activity breaks (dosage), *PSE* perceived self-efficacy, *sce* non-improvement in emotional and social school experience, *QOI* quality of implementation, *0* absent, *1* present;

Table 3 shows that 16 different configurations of the causal conditions can be found in the data set out of 2^5^ = 32 possible configurations.

**Table 4:** Intermediate Solution for Non-Achieving Emotional and Social School Experience

Solution	Raw Coverage	Unique Coverage	Consistency
qoi*kai	0.451	0.236	0.968
pab*pse*boi	0.214	0.044	0.984
QOI*pse*KAI	0.176	0.048	0.852
pab*qoi*pse	0.264	0.000	0.984
pab*pse*KAI	0.216	0.000	0.944

Abbreviations: *BOI* benefits of intervention, *PAB* physical activity breaks (dosage), *PSE* perceived self-efficacy, *QOI* quality of implementation;

Overall: solution coverage: 0.66; solution consistency: 0.94

Explanation: Capital letters (e.g. ‘PSE’) mean the causal condition is present; small letters (e.g. ‘pse’) mean the causal condition is not present

*represents the operator "AND" in the Boolean Algebra

Overall, the solution consistency was 94%, indicating that the same combinations of causal conditions consistently led to the same outcome. The solution coverage was 66%, revealing that 66% of all cases leading to non-success were described in the solution.

The first of these five recipes covering six cases and showing a high empirical relevance (raw coverage: 45%; unique coverage: 24%) was characterized by an unsatisfying quality of implementing the intervention and a low previous knowledge about and experience with the intervention. The second recipe showed that insufficient physical activity breaks (i.e. low dosage), associated with doubts regarding a teacher’s self-efficacy, and low expectations regarding the benefits of the intervention also did not lead to pupil improvement. A similar third recipe was characterized by an implementation process carried out with unsatisfying quality and quantity and associated with low levels of self-efficacy. The fourth recipe entailed a low dosage of physical activity breaks carried out with low levels of self-efficacy and high reported previous knowledge about the intervention. The fifth recipe was characterized by a satisfying quality of implementing the physical activity breaks, but carried out with low levels of self-efficacy and high reported previous knowledge about the intervention.

### Explaining pathways to success (occurrence of emotional and social school experience)

We identified t*eachers’ perceived self-efficacy* for implementing the intervention as a necessary condition (consistency: 88%, coverage: 56%) for achieving a positive emotional and social school experience in pupils. The other conditions did not emerge as necessary conditions. Overall, of the 32 logically possible combinations of causal conditions to explain success, 16 different combinations could be observed, revealing a high diversity of causal combinations (50% of the truth table rows were empirically observed, Table 5). Three different combinations of causal conditions met the minimum frequency threshold of one case and the minimum consistency threshold of about 0.8. The complex solution did not lead to any simplification as three solutions with five conditions each were described. For achieving the intermediate solution, contrary to assumptions for non-success, we supposed that the presence of the following four conditions could help explain the improvement of pupils’ emotional and social school experience: satisfying level of good quality and high quantity physical activity, high levels of teacher self-efficacy, and high expectations by teachers regarding the benefits in the intervention. The intermediate solution arrived at two different causal combinations to explain success with a coverage value of 52% and a consistency value of 81% (Table 6).

**Table 5:** Truth Table Summarizing Recipes for Achieving Pupils’ Emotional and Social School Experience

Conditions					Outcome	n	Consistency
PAB	QOI	PSE	BOI	KAI	SCE		
0	1	1	0	1	1	1	0.873
1	1	1	0	0	1	1	0.821
1	1	1	1	1	1	3	0.799
1	0	1	0	1	0	1	0.750
0	1	0	1	1	0	1	0.748
0	0	1	0	1	0	2	0.662
1	0	1	1	1	0	2	0.631
1	1	0	1	1	0	1	0.628
1	1	1	1	0	0	1	0.626
1	0	0	1	1	0	2	0.573
0	1	0	0	0	0	1	0.514
1	0	0	0	0	0	1	0.497
0	0	0	0	0	0	1	0.478
0	0	0	1	0	0	1	0.386
0	0	0	1	1	0	2	0.343
1	0	1	1	0	0	3	0.225

Abbreviations: *BOI* benefits of intervention, *KAI* knowledge about intervention, *n* number of school classes showing a specific causal condition, *PAB* physical activity breaks (dosage), *PSE* perceived self-efficacy, *SCE* improvement in school experience, *QOI* quality of implementation, *0* absent, *1* present

**Table 6:** Intermediate Solution for Achieving Higher Emotional and Social School Experience

Solution	Raw Coverage	Unique Coverage	Consistency
QOI*PSE*KAI	0.449	0.304	0.795
PAB*QOI*PSE*boi	0.213	0.068	0.880

Abbreviations: *BOI* benefits of intervention, *KAI* knowledge about intervention, *PAB* physical activity breaks (dosage), *PSE* perceived self-efficacy, *QOI* quality of implementation

Overall: solution coverage: 0.52; solution consistency 0.81

Explanation: Capital letters (e.g., “PSE”) mean the causal condition is present; small letters (e.g., “pse”) mean the causal condition is not present

*represents the operator "AND" in the Boolean Algebra

The first recipe associated with an improvement in pupils’ positive emotional and social school experience entailed the combinations of good quality implementation of physical activity breaks, implemented by teachers with a high self-efficacy, and a good previous knowledge about the intervention. This recipe presented a high empirical relevance with a raw coverage of 45% and a unique coverage of 30%. The second recipe consisted of the self-efficacious implementation of physical activity breaks in a high dosage and with high quality, but with a low teacher expectation regarding the benefits of the intervention.

## Discussion

We used the innovative method, fuzzy-set qualitative comparative analysis, to assess pathways to successfully achieving high SCE through a physical activity intervention for primary school children [34, 49]. To our knowledge, this method has not been used in the field of physical activity interventions before. The need to identify relevant points for improvement in the intervention itself or the implementation of the intervention was necessary, as the cRCT showed a null finding for changes in the primary outcome emotional and social school experiences [32]. We identified two pathways leading to success, where the most relevant solution highlighted the need for a high degree of teacher self-efficacy, prior knowledge, and good quality of intervention implementation. These results mirror the most important of the five pathways to non-success that emphasized an unsatisfying quality of implementing the intervention and a low previous knowledge about and experience with the intervention.

As the most relevant singular conditions appeared to be the quality and quantity of the implementation process, and teacher’s perceived self-efficacy. The latter could be identified as a necessary condition for success, meaning that it was present in most instances where the outcome occurred. In general, self-efficacy plays an important role in several behavioral change theories [29, 50] and other studies have supported the prominent role of raising the self-efficacy of intervention providers [51, 52] to support a good implementation of the intervention. Other important conditions were quality or quantity of the implementation process as these conditions were present in most identified recipes, revealing the conclusion of Durlak and DuPre’s systematic review [24] that better implementation leads to better intervention outcomes for participating individuals. In general, the quantity of the intervention implemented also plays an important role. Higher amounts of physical activity provided in the classroom positively influenced relevant outcomes of the intervention, such as BMI changes [53, 54]. To improve classroom behavior significantly, a daily 10- to 20-min active break condition may be necessary according to the results of a systematic review [17]. The five identified pathways to non-success revealed a high diversity of existing combinations of conditions. That QCA enables the detection of the existing causal complexity was also discussed in the evaluation of a veteran’s weight management program, where no condition pattern was shown by more than one organization [36].

Our study provided several implications for the improvement of the health promotion program *Classes in Motion* that may also be relevant for similar intervention programs: First, raising teachers’ self-efficacy and improving the quality of the teachers’ implementation process are important conditions leading to the desired outcome. As a physical activity intervention study showed that specific training can increase teachers’ perceived self-efficacy [55] we suggest adapting the teacher training. Holding different lessons consisting of “homework for teachers” and receiving immediate feedback from the trainers may contribute to the improvement of teachers’ self-efficacy and implementation of the intervention. Second, the participating teachers should be informed that regularly allowing a short break after 20 min of teaching is recommended [17] to achieve positive on-task behavior in children. Unfortunately, specific recommendations for achieving a positive class climate are missing, although one could argue that a class with focused and aware pupils would also report a better class climate. Third, for achieving the implementation of a certain amount of physical activity within the intervention, teacher logs could be used to sustain teacher motivation to implement the intervention, which is called the reactivity effect [56].

Our study has several limitations. The assessment of conditions, except quality of the implementation process, occurred only at the follow-up measurement. Therefore, potential changes over the course of the 1.5 academic year intervention period could not be detected. However, Smedegaard et al. [4] showed that the *Move for Well-being in School* intervention was relatively stable at three different time points during one school year. In the assessment of the conditions, we relied mostly on teachers’ self-report, which can be influenced by social desirability bias [26]. Furthermore, as no validated tools were available to measure the relevant conditions, we had to use self-constructed tools or adapt existing tools (e.g. self-efficacy [42]). Within the limited time frame of the evaluation, we used expert researchers in the field as well as the program’s primary stakeholders, such as the program leader and some trainers, to assess the tools’ face validity. Nevertheless, a full assessment process of the tools’ validity was not carried out. This may impede the generalizability of the results [30]. Future studies should rely on observations or interviews to achieve more reliable results [57]. Within our study, we applied a theoretical framework of implementation, the Consolidated Framework for Implementation Research, CFIR [29]. However, as the analysis process is restricted to a limited number of causal conditions (similar to a regression analysis), we could not address all relevant constructs or domains [40]. In particular, we could not consider any construct of *the inner setting* domain, describing organizational influences on the implementation process. Teachers rated the headmasters’ leadership engagement as high in all schools, therefore providing no variability and no explanatory power. In general, this factor was seen as important in other studies [26, 58]. We assumed that networks and communications, although important, may not have such an influence on the implementation as the individual teacher, since decisions for implementing programs were usually taken on an individual basis and not on a whole-school approach [59]. Nevertheless, we missed important barriers such as teachers’ lack of time for preparation, missing alignment with teachers’ goals, or teachers’ priority of academic subjects over physical activity in our study [4, 26, 27].

Within the course of the study, five teachers went on maternity leave. As this happened early during the first school year, the new teachers received most of the on-the-job training lessons. Therefore, we assume, that the change of teachers did not affect the results substantially. Overall, using fsQCA enabled us to sustain the fine-grained differentiation of the conditions although it required the definition of three different anchors per condition. We are confident that our in-depth knowledge of the cases when calibrating the data, due to additional qualitative interviews with the participating teachers and the inclusion of the knowledge of the stakeholders for developing the logic model, guaranteed valid results. Furthermore, while planning and conducting the study, we adhered to most standards of QCA such as justification for cases and keeping conditions at a moderate level [60]. We did not use X-Y plots for a graphical analysis of the overall solution, but used the text in the output. Overall, process evaluation can help focus on mediators to assess causal program theories [14], which is particularly central when a program failed to show success. Although this study focused especially on the implementation process and contextual factors, other reasons for not achieving the intended outcomes may have played a role [32]. FsQCA can be especially useful in that sense as it enables examination of causal complexity and it can identify different combinations of conditions that can lead to success and non-success, described as equifinality [35]. Although interaction effects can be assessed in regression analysis too, it’s becoming increasingly complex to explain them when assessing three or more variables. FsQCA can be used to analyze small to medium numbers of cases (e.g. 10 to 50) and is therefore especially useful when traditional statistical methods fail because of a small sample size. When applying a regression analysis to this data set, it would have been difficult to show statistically significant results due to the small sample size of 24 classes [35]. Overall, this analysis method identified two different pathways which led to success and five which explained non-success. It is a specific feature of this method as pathways identified for the presence of the outcome may not be similar to the pathways identified for the absence of the outcome [35]. This asymmetric concept of causation enabled us to study effective and ineffective implementation conditions, which was especially relevant as the intervention did not reveal the expected effects.

In the future, when using fsQCA, we recommend making the selection of the relevant conditions based on theoretical assumptions. To ease the selection process, we would suggest using a small-to-medium range theory [41] and focus only on a specific aspect of the implementation process [35]. Furthermore, reliable and validated instruments should be used for assessing the relevant constructs, if available.

## Conclusions

Different configurations of conditions measuring the implementation process and contextual factors helped to explain the success and non-success to improve *SCE* of the physical activity intervention *Classes in Motion*. Raising teachers’ self-efficacy and improving the quality of the teachers’ implementation process were important conditions to achieve the desired outcome. The program can be improved through better monitoring of the implementation of the intervention, regular feedback loops and structured learning opportunities for teachers, and clear recommendations regarding the dosage of the intervention. QCA has potential for an in-depth analysis of complex interventions implemented within organizations, as QCA can rely on detailed description of small to medium sample sizes, and can provide information which conditions lead to success or non success of a complex intervention.

## Additional files

Additional file 1: Logic Model. (GIF 74 kb)

Additional file 2: Questionnaire. (DOCX 40 kb)

## Abbreviations

%: Percentage
BOI: Benefits of intervention
C: Condition
CFIR: Consolidated Framework for Implementation Research
cRCT: Cluster randomized controlled trial
fsQCA: Fuzzy-set qualitative comparative analysis
KAI: Knowledge about the intervention
n: Number
O: Outcome
PAB: Physical activity breaks
PSE: Perceived self-efficacy
QCA: Qualitative comparative analysis
QOI: Quality of implementation
SCE: School-experience
SD: Standard deviation
