# Supplementary figures and images for: Pathways leading to success and non-success: a process evaluation of a cluster randomized physical activity health promotion program applying fuzzy-set qualitative comparative analysis
Source: BMC Public Health. 2018 Dec 18;18:1386. doi: 10.1186/s12889-018-6284-x (PMC6299632; doi:10.1186/s12889-018-6284-x)

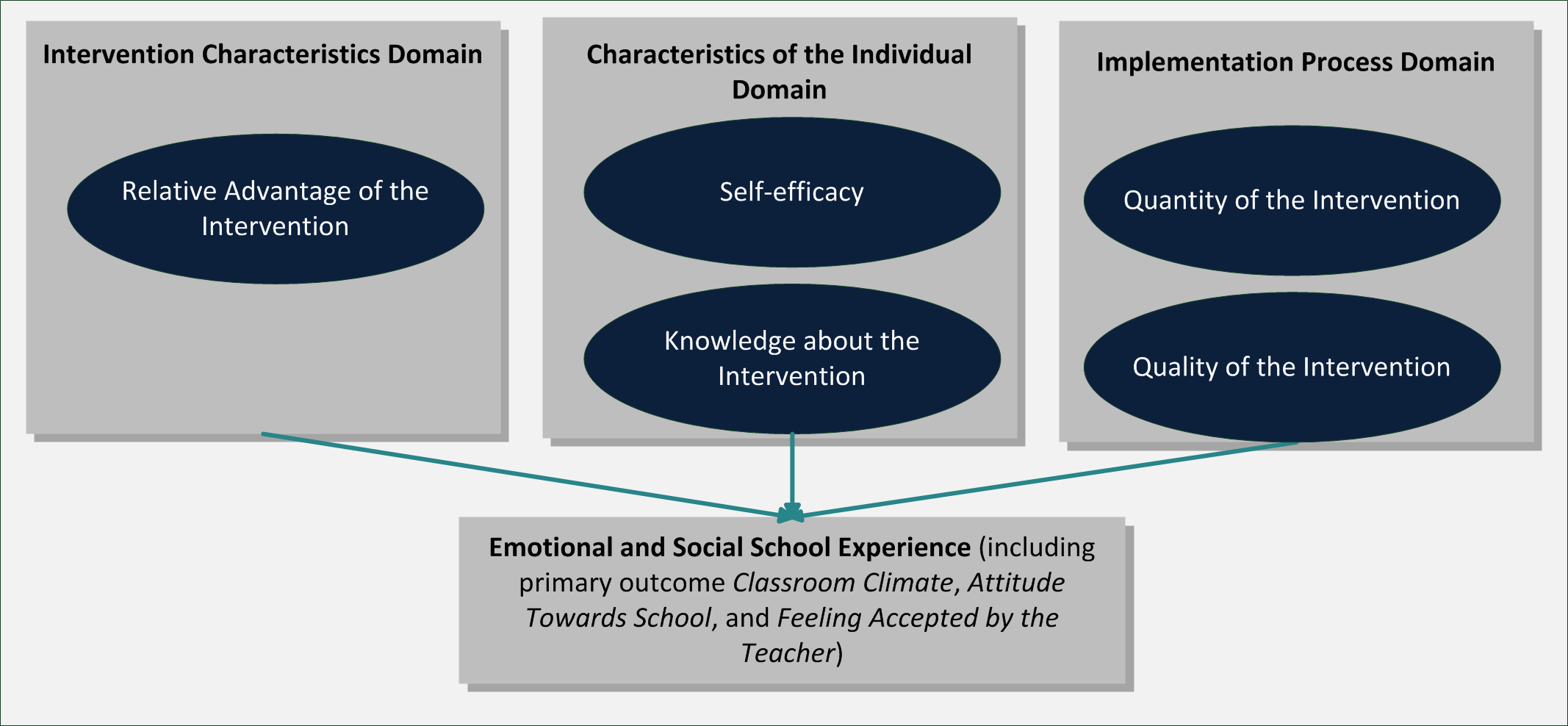

Supplement: Supplementary file 1 — Logic Model. (GIF 74 kb) [file 12889_2018_6284_MOESM1_ESM.gif]
