# Supplementary material for: Pathways leading to success and non-success: a process evaluation of a cluster randomized physical activity health promotion program applying fuzzy-set qualitative comparative analysis
Source: BMC Public Health. 2018 Dec 18;18:1386. doi: 10.1186/s12889-018-6284-x (PMC6299632; doi:10.1186/s12889-018-6284-x)
Supplement: Supplementary file 2 — Questionnaire. (DOCX 40 kb) [file 12889_2018_6284_MOESM2_ESM.docx]

**Additional File 2: Questions measuring the conditions**

The original questionnaire was used in German language. The translation into English is only done for transparency reasons.

*Physical activity breaks in minutes/school week (PAB):*

| **During the last month, how many minutes during classes have you used for active breaks on a daily basis?** (Please fill in!) | |
| --- | --- |
| during classes for active breaks | _________________ minutes/day |

*Teachers’ perceived self-efficacy of implementing the intervention (PSE):*

| **To what extent do you agree/disagree with the following statements?**  (For each line, please tick the appropriate box!) | | | | |
| --- | --- | --- | --- | --- |
|  | **strongly disagree** | **somewhat disagree** | **somewhat agree** | **strongly agree** |
| 1. I am confident that I can implement active breaks effectively. | □ 1 | □ 2 | □ 3 | □ 4 |
| 1. I possess the capacities and skills to implement active breaks. | □ 1 | □ 2 | □ 3 | □ 4 |

*Expected benefits of the intervention for pupils (BOI):*

| **To what extent do you agree/disagree with the following statements?**   - **Physical activity during classes …** (For each line, please tick the appropriate box!) | | | | |
| --- | --- | --- | --- | --- |
|  | **strongly disagree** | **somewhat disagree** | **somewhat agree** | **strongly agree** |
| 1. improves children’s concentration. | □ 1 | □ 2 | □ 3 | □ 4 |
| 1. induces children to disrupt the lessons. | □ 1 | □ 2 | □ 3 | □ 4 |
| 1. reduces children’s back problems. | □ 1 | □ 2 | □ 3 | □ 4 |
| 1. impairs the knowledge transfer. | □ 1 | □ 2 | □ 3 | □ 4 |
| 1. leads to an improvement of the class climate. | □ 1 | □ 2 | □ 3 | □ 4 |
| 1. distracts the children from concentration on teaching and learning. | □ 1 | □ 2 | □ 3 | □ 4 |
| 1. improves the children’s pleasure and motivation for learning. | □ 1 | □ 2 | □ 3 | □ 4 |
| 1. jeopardizes that children are equal to the requirements of the consecutive grade. | □ 1 | □ 2 | □ 3 | □ 4 |

*Knowledge about the intervention (KAI):*

| **To what extent do you agree/disagree with the statements on active breaks?**  (For each line, please tick the appropriate box!) | | | | |
| --- | --- | --- | --- | --- |
|  | **strongly disagree** | **somewhat disagree** | **somewhat agree** | **strongly agree** |
| 1. This topic was a major part of my education. | □ 1 | □ 2 | □ 3 | □ 4 |
| 1. I have already participated in further trainings on this topic. | □ 1 | □ 2 | □ 3 | □ 4 |
| 1. I have read books and teaching materials on this topic. | □ 1 | □ 2 | □ 3 | □ 4 |

**Comment:** The condition “*Quality of implementation”* wasn’t measured as a self-report question, but assed via trainers’ observation.
